# Factors Influencing the Accuracy of Infectious Disease Reporting in Migrants: A Scoping Review

**DOI:** 10.3390/ijerph14070720

**Published:** 2017-07-05

**Authors:** Paolo Giorgi Rossi, Flavia Riccardo, Annamaria Pezzarossi, Paola Ballotari, Maria Grazia Dente, Christian Napoli, Antonio Chiarenza, Cesar Velasco Munoz, Teymur Noori, Silvia Declich

**Affiliations:** 1 Interinstitutional Epidemiology Unit, AUSL Reggio Emilia, 42122 Reggio Emilia, Italy; annamaria.pezzarossi@ausl.re.it (A.P.); paola.ballotari@ausl.re.it (P.B.); 2 Arcispedale Santa Maria Nuova-IRCCS, 42123 Reggio Emilia, Italy; 3 National Centre for Epidemiology, Surveillance and Health Promotion, Istituto Superiore di Sanità, 00161 Rome, Italy; flavia.riccardo@iss.it (F.R.); mariagrazia.dente@iss.it (M.G.D.); christian.napoli@uniroma1.it (C.N.); silvia.declich@iss.it (S.D.); 4 Research and Innovation Unit, AUSL Reggio Emilia, 42122 Reggio Emilia, Italy; antonio.chiarenza@ausl.re.it; 5 European Centre for Disease Prevention and Control (ECDC), 17183 Stockholm, Sweden; cesar.velasco@isglobal.org (C.V.M.); teymur.noori@ecdc.europa.eu (T.N.); 6 IS Global, Barcelona Centre for International Health Research (CRESIB), Hospital Clínic, 08036 Barcelona, Spain

**Keywords:** migrant health, infectious diseases, surveillance, under-reporting

## Abstract

We conducted a scoping review of literature to improve our understanding of the accuracy of infectious disease monitoring in migrants in the Europe. We searched PubMed for papers relevant to the topic including: case reports, observational and experimental studies, reviews, guidelines or policy documents; published after 1994. We identified 532 papers, 27 of which were included in the review. Legislation and right to access health care influence both the accuracy of rates and risk measures under estimating the at risk population, i.e., the denominator. Furthermore, the number of reported cases, i.e., the numerator, may also include cases not accounted for in the denominator. Both biases lead to an overestimated disease occurrence. Restriction to healthcare access and low responsiveness may cause under-detection of cases, however a quantification of this phenomenon has not been produced. On the contrary, screening for asymptomatic diseases increases ascertainment leading to increased detection of cases. Incompleteness of denominator data underestimates the at-risk population. In conclusion, most studies show a lower probability of under-reporting infectious diseases in migrants compared with native populations.

## 1. Introduction

Migration is a heterogeneous phenomenon in Europe. At the beginning of the 20th century, many European countries saw large sections of their population emigrating both within and outside the current European Union (EU). This trend has changed over time with a growing number of EU countries becoming recipients of immigrants. Therefore, in the EU, there are countries with a long history of migration and others in which this is a recent phenomenon [1].

Some EU countries experience the effects of periodic exceptional inflows of new economic migrants or asylum seekers. Conversely, in other EU countries, migrants and their descendants have, over time, acquired a demographic and social stratification that might make their health profile closer to that of the host population [2]. As a result, the foreign-borne population between and within European countries is diverse in terms of countries of origin and length of stay. 

Migrants arriving to the EU are generally in good health [3]. This is due to several factors, such as good pre-travel health status and the fact that most infectious diseases have much shorter incubation periods than the time required to journey across the recognized Mediterranean migration routes. Notwithstanding, travelling conditions might make some migrants more vulnerable to health threats, due to exposures before arriving to the EU combined with low vaccination coverage. There is also evidence that migrants arriving and living in the EU and in the European Economic Area (EEA) are vulnerable to the negative effects on health of poor socio-economic living conditions [3].

EU/EEA Member States notify infectious diseases through a binding legal framework that defines a common standard for epidemiological surveillance [4,5,6]. However, concerns have been raised regarding the capacity of the existing surveillance systems to effectively monitor the health of migrant populations in the EU/EEA, and in particular infectious diseases [1,3,7,8]. A recent and extensive analysis of statutory infectious disease surveillance data in the EU/EEA, failed to draw overall conclusions about infectious disease burden among migrant populations in the EU/EEA [3] due to data limitations and differences in reporting between countries. A subsequent article [1] highlighted that these statutory surveillance systems lack variables to stratify cases of an infectious disease among migrant populations according to factors associated with an increased risk of contracting it. This in turn makes it difficult to interpret time trends and to identify which migrant population groups are most affected. In addition, syndromic surveillance systems targeting epidemic prone diseases have been developed alongside statutory systems to increase early detection and response capacities in countries experiencing large sudden influxes of migrants [9,10]. These parallel information sources, although timely, collect aggregated information on combinations of clinical signs and symptoms (i.e., syndromes) ahead of diagnosis and therefore cannot be imported into case-based, clinical and laboratory, national and EU surveillance systems [1]. 

**Figure 1:**
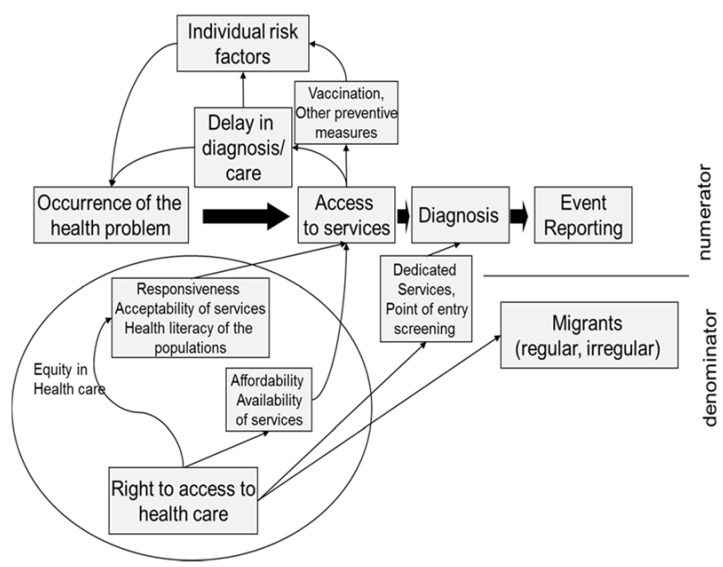
Interaction between migrant access to health care and indicators to monitor infectious diseases. In the middle, the theoretical flow from occurrence of the disease to reporting the event in a surveillance system is represented. Black arrows represent the causal effects of factors that could influence the probability of occurrence of the disease, access to health service, diagnosis, reporting, and computing a correct indicator of infectious diseases in migrants.

Three aspects are particularly relevant when addressing the issue of monitoring infectious diseases. Firstly, almost all our monitoring systems, particularly those focusing on infectious diseases, are based on the notification of identified cases of disease and other health related events (i.e., vaccination, infection status) that are diagnosed or occur only when a person has contact with the health services. Therefore, access to health services strongly influences any attempt to monitor the health status of a specific population. For a migrant, the probability of accessing health services is obviously influenced by the country legislation and the right for migrants to access health care, which could be different according to their legal status (i.e., regular, irregular, asylum seeker and all the other different conditions that the administrative system can distinguish). However, health system accessibility is also influenced by factors not directly linked to the laws regulating the right to access. These include: the affordability of the system (i.e., if it is free or not, if insurance is needed) and the responsiveness of health services (i.e., if mediators or language interpreters are available, accessibility of the clinics etc.) (Figure 1, bottom left part) [11]. 

Differential access to the health system could also change the probability of acquiring an infectious disease. For example, primary prevention in the case of vaccination or early treatment of contagious individuals/prophylaxis of contacts leads to the prevention of secondary cases (Figure 1, top left part). 

Secondly, there are different screening policies for infectious diseases targeting newly arrived migrants in the majority of EU/EEA countries [12] and in several non-EU countries in the Mediterranean Basin and Black Sea Regions [13]. In most countries, all newly arrived migrants or some specific subgroups undergo screening for tuberculosis (TB) [14,15,16,17], systematically increasing the probability of diagnosing the disease and/or latent infections (Figure 1, centre).

Thirdly, most of the indicators in a monitoring system need an accurate estimate of the at risk population, i.e., the denominator of the incidence and prevalence measures, to compare results in different geographic areas or in time (trends) and to allow meaningful interpretations of data. Knowing the immigrant population at risk has been recognised as a major problem in the field of migrant infectious disease epidemiology by the European Commission [5]. In fact, it is always difficult to understand if a reported case is included in the available denominators or not. This is even more difficult with respect to irregular migrants (Figure 1, right part). 

According to these considerations, we conducted a scoping review [18] to identify the factors influencing the accuracy of infectious disease monitoring in migrants in the EU/EEA.

## 2. Methods

### 2.1. Mapping the Scoping Review

Given the broad spectrum of possible mechanisms involved and the unknown number of factors acting through these mechanisms, we decided to conduct a scoping review. This kind of review allows identifying all the possible issues even if it will not give a systematic quantification of the effects. The choice of not giving quantitative effects is also justified because the magnitude of surveillance inaccuracy is country, time and disease specific [3], making the attempt to give a quantitative estimate not useful, while the underlying mechanisms can be common. 

We based our study design on previous European Centre for Disease Prevention and Control (ECDC) technical documents [3,19,20,21,22,23,24,25], on migrant health and infection, that were also based on scoping review studies [1].

We defined “Migrants” as persons, and family members, moving to another country or region to better their material or social conditions and improve the prospect for themselves or their family following the definition of the International Organization for Migration (IOM) [26].

After a preliminary search of the meta-literature, we designed a conceptual framework (Figure 1). This framework illustrates the process from the occurrence of the health problem to the reporting in a surveillance system, and all the factors that can differentially influence this process in the immigrant population compared to the native population.

The framework helped us to identify specific questions to formulate the PICOs (Population, Intervention, Comparison, Outcome) for the literature search. Thus the scope of this literature review can be summarized in the following questions:

- Which are the possible barriers in access to health services that cause under-diagnosis and other mechanisms that can affect the number of reported events (under-reporting, over-reporting, biases in reporting).
- What are the key issues with respect to the definition of the at risk population, i.e., the denominator, and how they affect the accuracy of infectious disease occurrence indicators in migrants and their comparability with indicators in native populations.
- How can screening programs for infectious diseases targeting newly arrived migrants introduce bias in event reporting.

### 2.2. Eligibility Criteria and Search Strategy

Articles were considered for inclusion if they: (i) were case report studies, descriptive and analytic observational studies, experimental studies, reviews, systematic reviews and meta-analysis, guidelines or policy documents (published and unpublished); (ii) were published after 1994; (iii) were published in English, French, Spanish, German or Italian; (iv) included data on infectious diseases; (v) included data from the EU/EEA. For misreporting and denominator, we decided to include also papers from Australia, New Zealand, Canada and United States, because these topics are not frequently treated by the literature and because they are the most methodological issues and less likely to be influenced by local peculiarities.

Three independent literature searches were conducted in PubMed, using both free text and Medical Subject Heading (Mesh) terms: one for the misreporting issues (under-reporting, over-reporting and biases in reporting); one for the denominator-related issues; and one for the effect of screening on reporting accuracy. 

The literature searches for misreporting and denominator were performed on 14 March 2014; the literature search for screening was performed on 17 March 2014, all records included in PubMed up that date were included.

The literature search strategies are presented in Supplementary Figures S1 and S2. Limiting the search to PubMed was considered adequate for this scoping review because it is aimed at identifying all the topics that are relevant for accuracy, but with the awareness that a precise quantification of this effect is not possible since it would be time, place, disease and population specific.

### 2.3. Study Selection and Data Collection Process

Eligibility assessment for inclusion was performed independently by two reviewers, initially by screening of all identified papers and reports based on title and abstract, excluding irrelevant papers, i.e., not mentioning accuracy or accuracy-related issues. All relevant articles were obtained in full text. 

### 2.4. Data Extraction and Synthesis

At the end of the search, the full texts were analyzed extrapolating the information about the study population (regular or irregular immigrant, refugee), the country, the health problem or diseases (see Supplementary Table S1). For all the papers, a narrative synthesis of the results was made. Efforts were made to make a synthesis of all the issues mentioned in the original papers. In some papers, a quantification of the inaccuracy (mostly under-reporting) was reported for migrant and native populations, allowing a comparison and sometimes an understanding of the determinants of the observed differences. In some cases, the objective of the included papers was not to assess the inaccuracy of reporting or explore its determinants, thus in these papers there were no explicit results but only reported indirect evidence of inaccuracy. In other cases, under-reporting was considered as a possible bias of the study and thus was only reported in the limitations section of the discussion without any quantitative assessment. Therefore, the results found in each paper were classified in three categories according to the grade of evidence reported: evidence of presence/absence of differential bias in reporting for immigrants and native population and sound quantification of this bias; evidence of differential bias but not quantifiable; hypothesis of presence/absence of differential bias in reporting. 

## 3. Results

### 3.1. Synthesis of the Literature Results: Misreporting

After search and selection, 16 papers were included, nine European and seven from other industrialized countries (Supplementary Figure S3). The studies covered the following diseases: tuberculosis (six articles); HIV and/or AIDS (two); hepatitis B or C (three); tuberculosis and HIV (one); pertussis (one), opportunistic infections (one), septic arthritis (one) and bacterial meningitis (one). Twelve articles focused on diagnosis; two on prevention; one on prevention, diagnosis and treatment and one on aspects unrelated to the phases of health care (i.e., self-reported health data). Four main issues were identified: over-reporting, under-reporting, other issues in accuracy of reporting, methods for surveillance.

#### 3.1.1. Possible Decrease of Under-Reporting

Four articles [27,28,29,30] reported less under-reporting in migrants than in the native population. In particular Melosini and coll [27], in a study conducted at a University Hospital in Central Italy, found lower TB under-reporting in migrants than in native Italians (18% of unreported cases in migrants vs. 68% in natives (*p* < 0.001); the authors could not explain this difference. Farchi and coll [29] found a reduction of TB under-reporting in the most recent period and a stronger improvement of TB surveillance among migrants (at the end of the period the unreported cases were 19% vs. 31% in natives). Nightingale and coll [28] suggested lower under-reporting of hepatitis B and C infection in migrant compared with native children, probably due to screening targeting newly arrived migrants. Finally, using a capture-recapture model, Giorgi Rossi and coll [30] found that being a migrant increased the probability of reporting bacterial meningitis. 

#### 3.1.2. Possible Increase of Under-Reporting

Jelastopulu and colleagues [31], describing TB incidence in western Greece, consider that over recent years there has been uncontrolled illegal migration from high TB endemic regions to many European countries, including Greece. The authors report that the majority of these migrants do not usually undergo any tuberculosis control programs and that possible cases among migrants are less likely to be diagnosed. They conclude that this could contribute to an underestimation of the disease burden. The authors did not perform any analysis of under-reporting in migrant populations, but only hypothesize this issue in the discussion.

Cohen et al. [32], in a Letter to the Editor, presented a chronic hepatitis B prevalence estimate in the USA, including high prevalence rates in migrant populations. The authors estimated the current burden of chronic hepatitis B in the USA to be approximately two million people. They concluded that an underestimation of the true number of infected individuals in the USA has occurred, mostly because the highest-at-risk populations are under-represented in surveillance studies, and a large percentage of chronically infected individuals remain undiagnosed.

Wohl et al. [33] discussed the under-reporting that may result from the use of English-based criteria for assessing mental status in the diagnosis of HIV encephalopathy among Spanish-speaking patients. 

Somerville et al. [34] found that infant pertussis hospitalisation rates in New Zealand are three to six times greater than rates in the USA, England and Australia. The hospitalisation rate varied with ethnicity, being higher for Maori and Pacific populations compared with European/other. The authors concluded that pertussis is under diagnosed, and consequently under-reported, in New Zealand, and that this phenomenon varied with ethnicity. 

#### 3.1.3. Other Issues in Reporting Accuracy

Two articles [35,36] focused on underestimation mostly as a confounder, with no clear mention of differential under-reporting by race/ethnicity. Ashrani and coworkers [35] addressed septic arthritis in males with haemophilia in the USA. The authors found that, compared with Caucasians, Afro-Americans/Hispanics/persons of other ethnic groups were more likely to develop septic arthritis. The authors suggest biological factors but also possible biases in diagnosis. Le Vu et al. [36] reported population-based HIV-1 new infection incidence in France. In this country, HIV transmission disproportionately affects certain risk groups. MSM (40%) were the most commonly diagnosed during recent infection, compared with French-national heterosexual women (28%) and men (22%), heterosexual non-French-national women (16%) and men (12%). The authors estimated under-reporting for each group, but did not present this result.

Watkins and colleagues [37], studying prospective Vietnamese migrants who had applied to migrate to Australia, found discrepancies between self-reported data collected in the confidential interview and the medical screening for an Australian visa. The authors argued that these discrepancies indicate that prospective migrant reports of health status and health behaviours may be biased by expectations that unfavourable responses could adversely affect their chances of migration.

Porta et al. [38] and Winston et al. [39] reported the risk of misclassifying route of infection and other exposures in HIV and TB, respectively [38,39]. Rose et al. [40] estimated the contribution of HIV infection to the recent rise in TB in England and Wales. The authors concluded that as HIV infection may be undiagnosed in patients with TB, and TB may be unreported in patients with diagnosed HIV infection, the true extent of co-infection would have been underestimated and that this phenomenon was stronger for some ethnic minorities.

#### 3.1.4. Methods in Incidence**/**Prevalence Estimates

Three articles [29,41,42] aimed to calculate an unbiased infectious disease prevalence estimate for the total population adjusting for high prevalence and including some risk groups, especially migrants, which are usually under-represented in surveys.

Marschall et al. [41] aimed to calculate an adjusted prevalence of chronic hepatitis B estimate for the total host population in The Netherlands, including migrants and other risk groups. These risk groups were not well represented in a previous representative sero-surveillance study (from 1995), which probably led to an underestimation of the true HBsAg prevalence. 

Baussano and coworkers [42] aimed to assess the completeness of the TB registration systems and estimation of TB incidence and under-reporting in a low-prevalence area (Piedmont Region of Italy). Sensitivity of the notification system was estimated to be 77 (95% CI 74–79) for Italians and migrants from low-prevalence countries and 86 (95% CI 81–90) for migrants from high-prevalence countries. Farchi et al. [29] found 39% under-reporting of TB in another Italian region, Lazio. The overall estimated TB incidence rate was 16.7 cases per 100,000 population (95% CI 16.3–17.3), varying according to the subset investigated: 12.7 for individuals from low TB prevalence countries and 214.1 for migrants from high TB prevalence countries. The authors concluded that there are inaccurate estimates of the annual incidence of TB, particularly among high-risk subsets of the population such as immigrants from high TB prevalence countries.

### 3.2. Synthesis of the Literature Results: Denominators

Out of the four articles included, three were on TB and one on HIV, two European and two from other industrialized countries (Supplementary Table S1).

#### Missing or Estimated Denominator Data

Farchi et al. ([29], initially extracted in the misreporting research) and Odone et al. [43] described differences in diagnosed TB cases between foreign born and native populations in Italy. Both found that the incidence in the foreign-born population is much higher than in the native-born population. However, it was impossible to accurately estimate the incidence of TB, due to lack of a valid denominator. In fact, residence permits are unanimously considered an underestimation of the number of foreign people actually living in Italy because they do not include irregular migrants. On the contrary, notified infectious diseases also include cases among irregular migrants. There is no way to distinguish between cases of disease among regular and irregular migrants on the basis of the Italian statutory system for infectious disease surveillance. Odone et al. [43] tried to compute a TB incidence rate taking into account an estimated 20% (10% in 2003) of not regularly registered migrants. The authors admit that it is a very rough estimate as data on irregular migrants are unavailable. In addition, by distinguishing cases on the basis of the country of birth, the authors could not include young foreigners who were born in Italy in the migrant sub-group.

Das and colleagues [44], analysing the epidemiology of tuberculosis in New Zealand, explained that for the calculation of the TB incidence rate by ethnicity, Statistics NZ prioritised an ethnicity approach for both the numerator (reported cases) and denominator (at risk population). However, it is possible that the numerator (surveillance data) and denominator (census data) were collected differently. In the census, ethnicity recorded is self-identified. Hospital records are more frequently coded with sole rather than multiple ethnicities. These practices create a numerator-denominator mismatch, which cannot be eliminated. 

Marc et al. [45] analysed HIV trends among Haitian-born persons in the United States. The authors discussed the importance of having accurate denominators to estimate rates of new HIV infections in the Haitian population. Using estimates from the 2007 American Community Survey, the results suggested a seven-fold over-representation of Haitians in the CDC AIDS surveillance data. In contrast, using denominator estimates from the Haitian Consulates, Haitian-born persons in the US, at this time, had similar AIDS rates to African Americans overall, which challenges beliefs that Haitian immigrants have a higher prevalence of AIDS than other groups.

### 3.3. Synthesis of the Literature Results: Screening

Nine papers included in the screening search tackled the issue of disease reporting inaccuracy (Supplementary Table S1), eight were on TB while one article focussed on HBV and HCV. 

Arshad and coll. [46], in a systematic review and meta-analysis of screening yield for TB, found a higher yield for refugees than for regular migrants and for asylum seekers, probably because the refugees are not self-selected to be healthy, i.e., no healthy migrant effect. The prevalence in migrants was also found to be higher than the prevalence in their countries of origin. The authors suggested that this could be due to problems of self-selection of high risk people among migrants. However, over-reporting in screening or under-reporting in national statistics was also suggested to interpret these results.

Pace-Asciak and coworkers [47] described the results of mandatory screening of TB at entry point and subsequent surveillance of all migrants from 2002 to 2005 in Malta. The authors found a very high TB prevalence at entry and cases with onset in the first months after entry. Surveillance was found, however, to possibly overestimate incidence and prevalence because the total number of undocumented migrants was under-estimated. Screening at entry was found not to reduce diagnostic delay for cases diagnosed after entry. This suggests firstly that barriers to access services at community level also exist when screening is offered free of charge. Secondly, that screening did not lead to overestimating incidence *per-se.*

Verver et al. [48] found that TB cases detected through screening were less likely to be the first cases of a cluster. The authors highlighted the risk of confounding with length of stay in The Netherlands, that is shorter for screen-detected cases and influences the probability of infecting other people.

Erkens et al. [49] described the routine TB screening in The Netherlands for newly arrived migrants, about 70,000 individuals followed for 29 months after arrival. The yield of screening was high in the first year after arrival and related to the prevalence of disease in the country of origin of migrants. 

Fernández Sanfrancisco et al. [50] described the prevalence of TB infection in migrants from different African countries at the Calamocarro refugee camp in Ceuta, Spain, and described the mis-classification of old prevalent cases as incident cases in a cross-sectional study with screening-like procedures.

Pareek and colleagues [51] conducted a study on TB screening of migrants in UK. Considerable heterogeneity and deviation from national guidance were found to exist throughout the UK new entrant screening process. Screening for latent TB detected more cases than screening for active TB and with high TB burden primary care centres undertaking the least screening and detecting fewer cases. 

Kruijshaar et al. [52] found that TB incidence among immigrants in UK is not directly associated to the prevalence of TB in origin countries. The authors suggest that this could also be due to different screening policies at arrival. 

Diel et al. [53], in an article on the epidemiology of TB in Hamburg, described that only a minority of TB cases among migrants was found by screening asylum seekers and suggested no difference with other migrants that are not routinely screened.

Nightingale et al. [28], in New South Wales, initially extracted in misreporting research, found over-reporting of HBV infection for foreign children. In particular, most of the children with HBV infection were found to be either refugees/migrants, or children of refugees/migrants. Since refugees are often screened after arrival in Australia, the authors interpreted these results as due to an ascertainment bias in this group. 

Table 1 summarizes the main determinants of surveillance accuracy found in the literature search.

### 3.4. Risk of Biases of the Included Papers

In this scoping review it was impossible to make a formal appraisal of the quality of studies and probability of biases, because we focused on an aspect that was marginal for the authors of the original studies. From the original paper authors’ point of view, misreporting was often only a possible source of bias in measuring the association that was the main objective of their study. Indeed, some of the papers included in the review only reported hypotheses on which unmeasured biases could affect their estimates of disease occurrence in migrants and native population. We tried to categorise the quality of evidence with an explicit grading. Out of 16 studies included in the search about misreporting, four [27,29,30,42] reported evidence of a quantifiable inaccuracy of infectious disease reporting, three reported an ascertained but not quantifiable inaccuracy of reporting [28,32,34], while the other nine reported only a possible inaccuracy of reporting [31,33,35,36,37,38,39,40,41]. For the search about denominators all four papers reported ascertained but not quantifiable inaccuracy of reporting [29,43,44,45]. Finally for the search about screening, four papers reported an ascertained but not quantifiable inaccuracy of reporting [28,46,48,49], while the other five reported only a possible inaccuracy of reporting [47,50,51,52,53] (Table 1). 

## 4. Discussion

### 4.1. Limits

The search strategies tried to identify all the papers reporting direct/indirect evidence or hypotheses about factors affecting the accuracy of infectious disease reporting in migrants. Most of the papers identified did not focus on our specific topic and only treated it as a possible bias of their results. We cannot exclude that, in the search process, we missed some papers because the misreporting or the biases were not mentioned in the title or abstract.

Even if the topic of the review is infectious diseases, we retrieved papers mostly on TB. For screening, this cannot be considered a bias but simply reflects that TB is the only disease for which there are guidelines recommending screening [14,15,16,17]. For the misreporting, this is actually a limit of our review determined by the scarcity of literature on other diseases. 

Only four papers were included in the search of the denominator-related issues. We cannot exclude that other papers on migrant health, not specifically referring to infectious diseases, have tackled the problem of estimating correct denominators, particularly of undocumented migrants, with possibly interesting insights. Nevertheless, we decided to keep the search focussed on infectious diseases because, to our knowledge, the infectious diseases surveillance systems are the only ones in which non-resident migrants, regular or irregular, can be included in the numerator but not in the denominator. The main source of inaccuracy of infectious disease occurrence in immigrants is this peculiar mismatch between numerators and denominators that is not present in other health information sources, such as mortality or hospitalization discharge, where the residence is well reported in the numerators.

Only for the issues of misreporting and denominators we included in our search studies from non-European industrialised countries. The problems emerged from non-European industrialised countries were similar in the case of denominator: possible under-estimation of the true denominator and mismatch of denominator and numerator. For the factors leading to misreporting, some authors from non-European countries focussed on biases due to survey methods [32,33,35] or administrative procedures [37] that are not commonly used in Europe.

Another limit of this review is that we considered the immigrants as single population, simplifying the fact that they are a heterogeneous group characterised by different histories of migration, different countries of origin and different length of stay in the host country. When evidence was available, we highlighted in our findings whether the observations concerned newly arrived or immigrants with any length of stay; immigrants from a specific country of origin or mixed populations. As the purpose of this review was to provide an overall understanding of the drivers of over/under-reporting in migrant populations rather capture the entire complexity of the phenomenon, we do not think that this simplification undermines the findings.

### 4.2. Main Findings

This review analysed several factors affecting the accuracy of reporting infectious disease in migrants. Both factors favouring both under- and over-reporting were mentioned by the literature (Table 1). Surprisingly, despite the *a priori* concern mostly being about the difficulty to detect and report infectious diseases in migrants, most of the papers found evidence or insights pointing towards lower under-reporting in migrants than in native populations [27,28,29,30]. 

**Figure 2:**
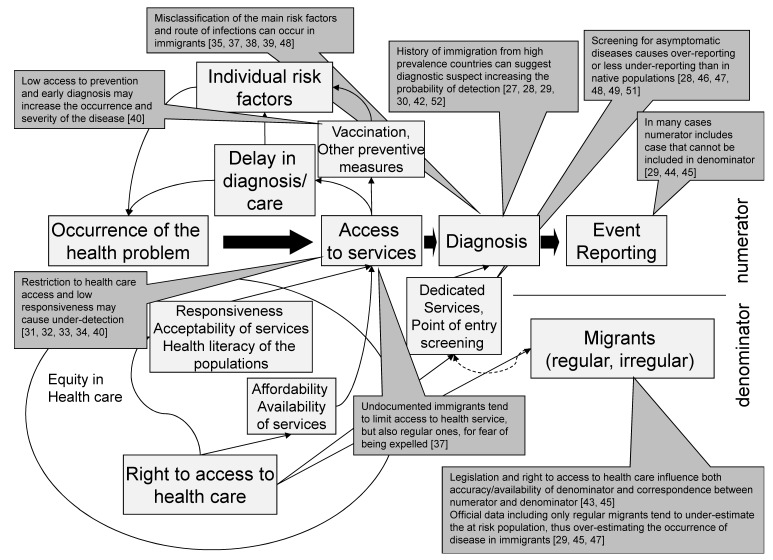
Results of the scoping review reported on the conceptual framework of the interaction between migrant access to health care and indicators to monitor infectious diseases. In the middle the theoretical flow from occurrence of the disease to reporting the event in a surveillance system is represented. Black arrows represent the causal effects of factors that could influence the accuracy of infectious disease surveillance in immigrants. The grey boxes report the results of the literature review.

The main factors contributing to this phenomenon are screening for infectious diseases in newly arrived migrants, in particular for TB [28,46,47], and higher clinician attention to infectious diseases when examining people with a history of migration [27,51]. Finally, difficulties in correctly estimating the population at risk, in most cases, lead to an underestimation of the migrant population and consequently to an overestimation of incidence and prevalence rates of infectious diseases [29,43,47]. On the other hand, the main reason for under-reporting has been linked to the barriers to health service access experienced by many migrants [31,43]. Several authors pointed out that the most relevant barrier for migrants to access to health services, and thus to infectious disease reporting, is the fear of being identified as irregular, even when enjoying a regular status [37]. Figure 2 tries to put the findings of this scoping review in the context of the conceptual framework we initially adopted. 

Not all the possible effects found in the literature have the same level of evidence. In fact, as reported in Table 1, in most cases factors determining an increased under-reporting in migrants are only theoretically hypothesized by the authors, while the factors determining lower under-reporting in migrants, such as entry screening and higher attention in diagnosis and notification, have been ascertained and, in some cases, also quantified. The other factor leading to an over-estimation of the risk of disease in migrant compared to native, i.e., the under-estimation of the denominator and the inclusion in the numerator of people that are not in the denominator, has also been ascertained. 

### 4.3. Implication for Surveillance Practice

Our results can give some suggestions for improving existing surveillance systems and to design a European network of surveillances:

- We should focus on improving the existing systems for native and migrant populations alike, with specific attention to under reporting in native populations (in particular for TB and meningitis). We believe this approach, as opposed to establishing special surveillance systems for migrant populations, would provide a better picture of the actual epidemiology of these diseases.
- Surveillance systems should be better characterized, through the inclusion of specific surveillance variables, the main risk factors for infectious diseases among migrants, i.e., country of origin [46,49,51], length of stay [48], and history of migration [46,52] in order to better understand the observed trends and plan public health interventions.
- Finally, some of the most relevant biases in estimating disease occurrence could be avoided providing a certain indication about the presence or not of the case reported in the available denominators for foreigners [29,43,44,45]. In most cases it would be sufficient to include a variable on the resident status of the foreigner (i.e., if he/she is formally resident in the host country or not, without any further assessment of regular or irregular state). This would allow to calculate unbiased rates and to make comparisons between different groups of immigrants or with the native population.

## 5. Conclusions

Even if barriers in access to health services have been observed by several authors and these barriers could decrease the probability of disease reporting, almost all quantitative evidence shows a lower probability of under-reporting infectious diseases in migrants than in native populations. These conclusions are counterintuitive because public health experts expect to know more on disease occurrence among native populations in their countries than in migrants. When interpreting available data from infectious disease surveillances, public health operators should consider that figures on disease occurrence in migrants are probably closer to the real occurrence than the same figures for the native population. 

## Figures and Tables

**Table 1:** Summary of results.

Issue	Main Findings	Type of Evidence	References
Misreporting			
Decrease under-reporting	TB and meningitis are more often reported in migrants	Certain and quantified	[27,29,30,42]
	Improvement in TB surveillance was stronger for immigrants	Certain and quantified	[29]
Increase under-reporting	Illegal immigrant could be under-diagnosed for TB	Possible	[31]
	Lower response in surveys could under represent immigrants	Certain not quantified	[32,41]
	Language barriers decrease probability of syndromic diagnoses	Possible	[33]
	Under-diagnosis in minorities	Possible	[40]
	Under-diagnosis in minorities	Certain not quantified	[34]
Other effects	Biases in systemic infection diagnosis	Possible	[35]
	Biases in reporting information	Possible	[36,37]
	Inaccuracy about the route of infection for TB and HIV	Certain not quantified	[38,39]
Denominator	Under-estimation of the real at-risk population for immigrants	Certain not quantified	[29,43]
	People included in the numerator are not always part of the denominator	Certain not quantified	[29,43,44,45]
Screening	Screening increases the probability of diagnosis	Certain and quantified	[28,46,47,48,49,51]
	Misreporting of prevalent cases as incident	Possible	[50]
	Screening modalities and implementation impact on detection	Certain and quantified	[51]
	Screening could leave less cases to be detected in the routine surveillance	Possible	[52]
	Screen-detected cases are less often first cases of a cluster	Certain and quantified	[48]
	Small effect on overall incidence	Certain not quantified	[53]

## Supplementary Materials

The following are available online at www.mdpi.com/1660-4601/14/7/720/s1, Figure S1: Search strategy for Misreporting or Denominators; Figure S2: Search strategy for Screening; Figure S3: Synthesis of the literature results; Table S1: Extraction table. Included papers.
