# Supplementary material for: Factors Influencing the Accuracy of Infectious Disease Reporting in Migrants: A Scoping Review
Source: Int J Environ Res Public Health. 2017 Jul 5;14(7):720. doi: 10.3390/ijerph14070720 (PMC5551158; doi:10.3390/ijerph14070720)
Supplement: Supplementary file 1 [file ijerph-14-00720-s001.pdf]

Supplementary Materials

Factors Influencing the Accuracy of Infectious Disease Reporting in Migrants: A Scoping Review

Paolo Giorgi Rossi, Flavia Riccardo, Annamaria Pezzarossi, Paola Ballotari, Maria Grazia Dente, Christian Napoli, Antonio Chiarenza, Cesar Velasco Munoz, Teymur Noori and Silvia Declich

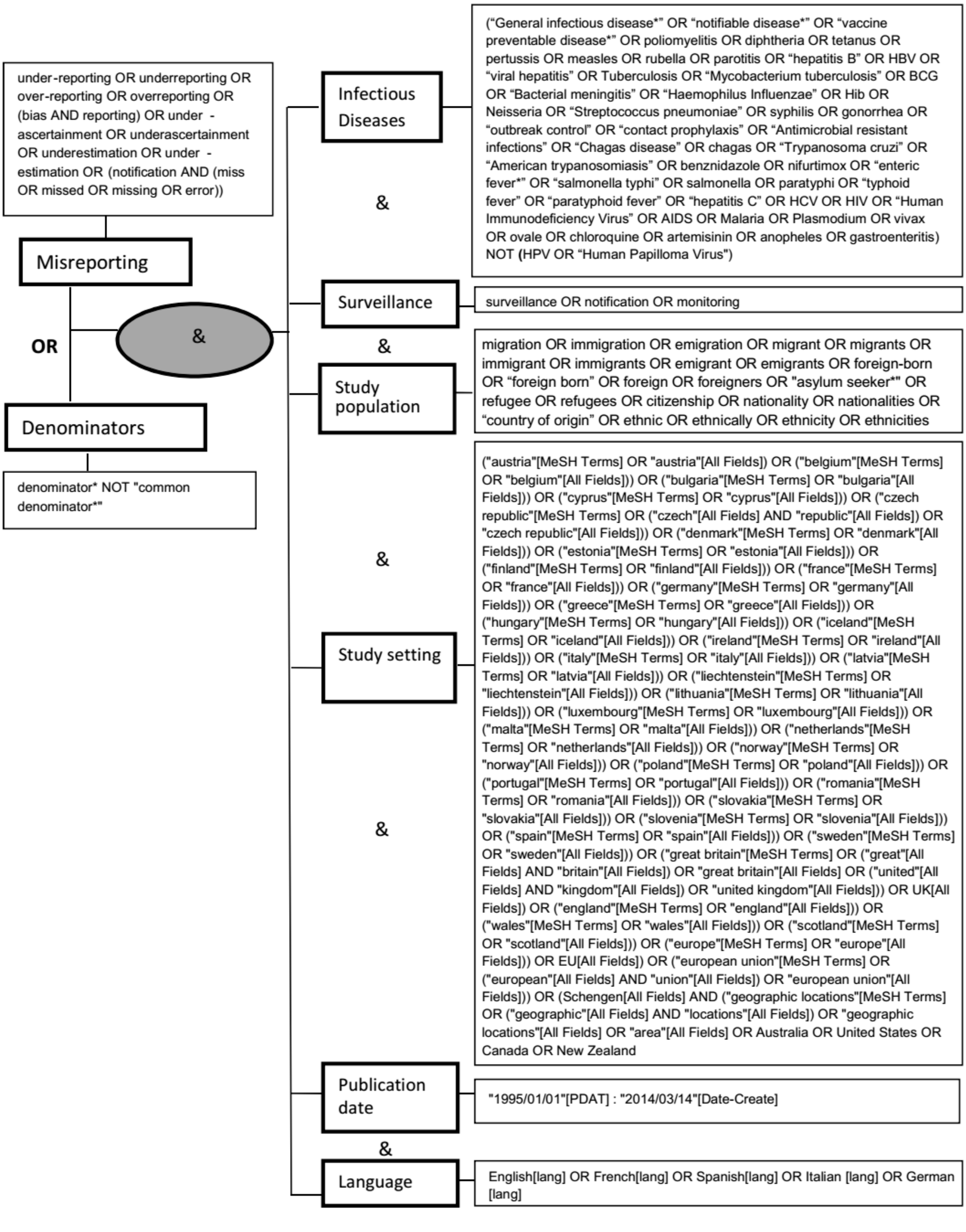

Figure S1. Search strategy for Misreporting or Denominators.

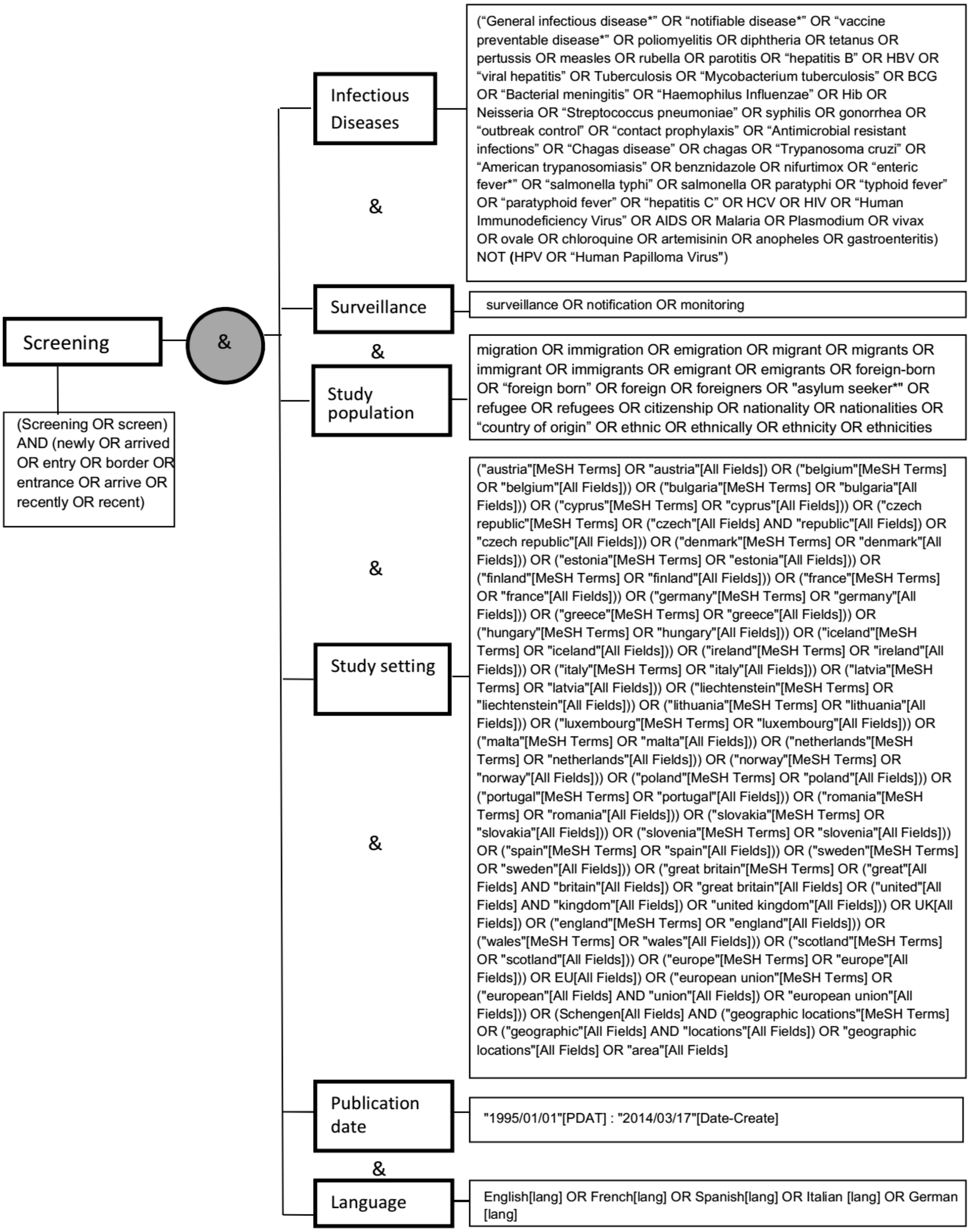

Figure S2. Search strategy for Screening.

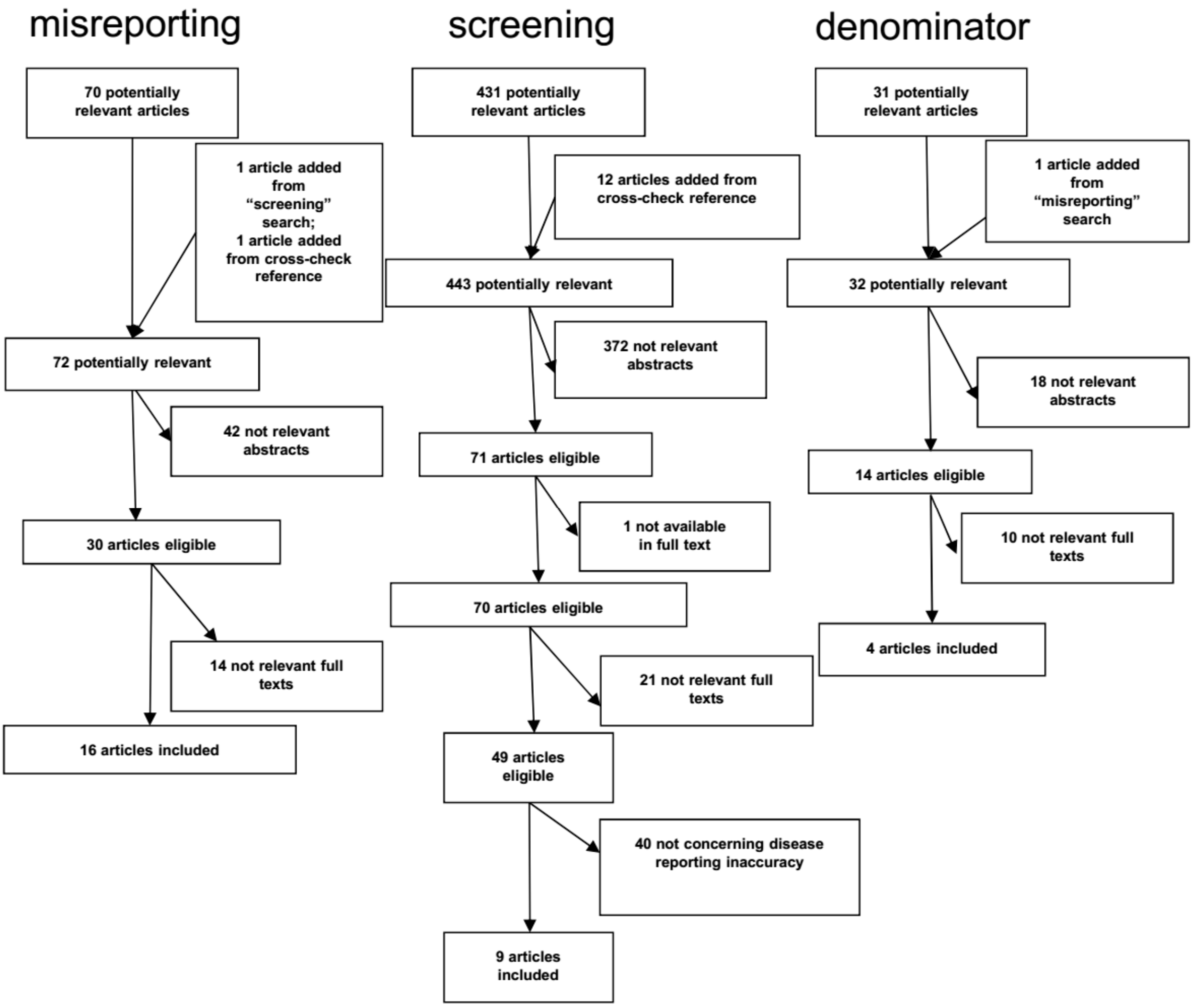

Figure S3. Synthesis of the literature results.

Table S1. Extraction table. Included papers.

First author and year	Country; disease	Population; type of migrant	Reported outcomes	Author's conclusion
**Misreporting**				
Melosini 2012	Italy; tuberculosis (TB)	general population attending hospital; foreign citizens	TB underreporting	TB is underreported in Pisa, particularly in older patients and those without microbiological confirmation. The TB code in first position of HDR seems fairly accurate in confirming TB diagnosis
Nightingale 2009	Australia; hepatitis B or hepatitis C	<18 years; born overseas (ie foreign-born)	characterisation of epidemiological, clinical and laboratory features of children with chronic hepatitis B (HBV) or C (HCV) infections.	Although advanced liver disease was uncommon in children referred with HBV or HCV infection, a large number of infected children in NSW were not referred for specialist medical care, indicating that opportunities to intervene early in the natural history of these infections, particularly HCV, are being missed.
Giorgi Rossi 2009	Italy; bacterial meningitis	four sources : hospital surveillance of bacterial meningitis, laboratory information system, the mandatory infectious diseases notifications, and hospital information system; immigrants	reduction of underreporting	Vaccine policies should be planned and monitored based on these results. The integrated surveillance system allowed us to observe a drop in H. influenzae b meningitis incidence consequent to the implementation of a mass vaccination of newborns.
Jelastopulu 2009	Greece; TB	all population, immigrants and refugees	incidence rates	The study demonstrated a substantial underestimation of TB burden in West Greece. In the face of the massive influx of immigrants and refugees coming from regions with high TB incidence and the increase of the number of drug-resistant cases a reliable and complete notification of TB is crucial in the planning of programs and development of appropriate control policies.
Cohen 2008	USA; hepatitis B	all population; immigrants, in particular Asians and Pacific Islanders (API).	prevalence	Chronic hepatitis B remains an under-recognized disease in the USA The nation needs to support public health surveillance systems that yield reliable state and local data and can be used to calculate more accurate estimates of chronic hepatitis B. The Hepatitis B Foundation calls for action to be taken to allow all individuals in the USA who are chronically infected with hepatitis B to be counted.

Whol 2003	USA; opportunistic infection	all population; Mexican-born and central American-born latinos	compare opportunistic infection (OI) rates for U.S.-born, Mexicanborn, and Central American-born Latinos in the pre- or post-highly active antiretroviral therapy (HAART) era.	In addition to underreporting that may result from the use of English-based criteria for diagnosing HIV encephalopathy among Spanish-speaking patients, these active antiretroviral therapy (HAART) era data suggest that variation in opportunistic infection (OI) risk among Latinos may also be explained by acculturation factors, such as loss of social support systems and negative lifestyle changes.
Somerville 2007	New Zealand; pertussis	infants <12 months old, hospitalised with pertussis; ethnicity	incidence; pertussis disease burden.	Infant pertussis hospitalisation rates are three to six times greater than rates in the USA, England and Australia. Underestimation of disease burden by passive notification in hospitalised infants is modest, suggesting a high degree of clinical awareness by paediatricians in NZ. New immunisation strategies are needed to protect infants from a younger age.
Rose 2002	England and Wales; TB, HIV/AIDS	16–54 years old at diagnosis of tubercolosis on 1993 or 1998; black african, indian subcontinent (India, Pakistan, Bangladesh)	association between tubercolosis and HIV infection	In 1998 the number of tubercolosis patients co-infected with HIV in England and Wales had nearly doubled since 1993, with most of the increase occurring in London. Routine HIV testing of all patients with tubercolosis should now be considered, particularly in patients of white or black African ethnic origin under 55 years of age.
Ashrani 2008	USA; Septic arthritis	Males; race/ethnicity	incidence, risk factors and impact of septic arthritis.	Septic arthritis is an uncommon complication of haemophilia occurring primarily in joints most affected by bleeding and reparative surgical interventions.
Farchi 2008	Italy; TB	all population; immigrants, foreigners, foreign-born	TB incidence	Despite a decreasing trend, TB incidence is still over 10/100 000 in Lazio region. Targeted interventions for immigrant populations are essential for controlling TB.
Le Vu 2010	France; HIV	all population; foreign citizens	incident HIV diagnoses	In France, HIV transmission disproportionately affects certain risk groups and seems to be out of control in the MSM population. Incidence should be tracked to monitor transmission dynamics in the various population risk groups and to help to target and assess prevention strategies.
Watkins 2005	Australia; TB	> 15 years old; prospective Vietnamese migrants who had applied to migrate to Australia	association between self-reported health status and several commonly used clinical indicators of health.	These findings support the validity of self-reported health status assessment among prospective migrants, although the assessment of subjective indicators of health during compulsory medical screening may be limited by reporting bias.

Marschall 2008	Netherlands; hepatitis B	all population; first generation migrants and second-generation migrants	prevalence; importance of targeting migrants and their close contacts adequately in screening programmes, vaccination and treatment for chronic hepatitis B.	Our results show the high importance of targeting migrants and their close contacts adequately in screening programmes, vaccination and treatment for chronic hepatitis B.
Baussano 2006	Italy; TB	all population; immigrants	to assess the completeness of the TB registration systems and estimation of TB incidence and under-reporting.	When multiple recording systems are available, record linkage and CR analysis can be used to assess TB incidence and the completeness of different registers, contributing to a more accurate surveillance of local TB epidemiology.
Winston 2012	USA, TB	<18 years; migrants analysed by place of birth; I and II generation	prevention	Three-quarters of pediatric patients with TB in the United States have potential TB exposures through foreign-born parents or residence outside the United States. Missed opportunities to prevent TB disease may occur if clinicians fail to assess all potential TB exposures during routine clinic visits.
Porta 2004	Italy; HIV	all population; foreign-born	analysis of temporal trend of HIV infection.	HIV infection is increasing in women and immigrants; people are being infected more frequently by sexual transmission than intravenous drug use.
**Screening**				
Arshad 2010	many countries; TB	new immigrants at the point of entry; refugee, regular, asylum seeker	screening yield	These results provide useful data to inform the development of coherent policies and rational screening services for the detection of immigrant-associated tuberculosis.
Pace-Asciak 2013	Malta; TB	undocumented immigrants to Malta; undocumented boat immigrant	TB prevalence; screening coverage; yield at fist and subsequent screening	Tailoring TB control strategies to this migrant population is essential for TB control in Malta. Awareness of increased risk of TB needs to be ongoing, not just at entry but for many years after arrival, even in resettlement countries.
Verver 2002	The Netherlands; TB	all TB cases notified; foreigners from high prevalence countries	Risk of being the first case in a cluster	Screening of immigrants can reduce tuberculosis transmission. This effect is in part due to confounding by duration of stay.
Erkens 2008	The Netherlands; TB	all immigrants from non western countries entering from 1998 to 2002; all new entries.	Yield of screening; TB prevalence at entry.	Yield of entry screening was high. Overall coverage and yield of follow-up screening was low. Follow-up screening of immigrants with a normal chest radiograph from countries with an incidence of <200 per 100,000 individuals was therefore discontinued.

Fernández Sanfrancisco 2001	Spain; TB	Population: immigrants, 89.0% being males and 11.0% females, respectively averaging in age 24.9 +/- 4.3 and 23.4 +/- 4.1; immigrants from 36 African countries.	Prevalence of tuberculosis among the immigrant population.	The immigrant population from central African countries shows a higher prevalence of tuberculosis infection, comprising a group at risk of contracting this disease. Therefore, it is of fundamental importance to implement specific programs to actively detect tuberculosis infection during their stay in our city, taking advantage of their being grouped together upon entry into our country, prior to their final emigration to localities throughout the mainland and subsequent mixing among the host country population.
Pareek 2011	UK, TB	all population; all migrants.	Investigate how UK primary care organisations (PCOs) screen new entrants and whether this differs according to TB burden in the PCOs (incidence <20 or ≥20 cases per 100,000 per annum).	Among PCOs screening for LTBI, there was substantial deviation from national guidance in selection of new entrant subgroups and screening method. Considerable heterogeneity and deviation from national guidance exist throughout the UK new entrant screening process, with high-burden regions undertaking the least screening. Forming an accurate picture of current front-line practice will help to inform future development of European new entrant screening policy.
Kruijshaar 2013	UK, TB	Population: immigrants in UK. Type of migrant: top 10 for TB incidence in England and Wales.	Number Need to Screen by each country of origin.	Targeting screening on the basis of incidence in the UK may improve effectiveness.
Diel 2004	Germany, TB	all residents; asylum seekers and foreign born <5 or >=5 years resident.	cluster vs non cluster TB cases	In conclusion, only a minority of TB cases among foreign-born individuals are detected by screening of asylum seekers or conventional contact tracing. Recent transmission does not play an important role in TB among immigrants in Hamburg. Authors claim for screening with TST also other immigrants.
Nightingale 2009	Australia; hepatitis B or hepatitis C	<18 years; born overseas (ie foreign-born)	See above in Misreporting	See above in Misreporting.
**Denominator**				
Odone 2011	Italy; TB	all population; not Italy-born	to investigate the epidemiology of tuberculosis (TB) in not Italy-born compared to Italy-born cases	In low-incidence regions, prevention and control of TB among sub-groups at risk such as the foreign-born population is a matter of public health concern. In addition, increasing immigration rates may affect TB epidemiology. TB among immigrants is characterized by particular clinical features and

				risk factors, which should be analyzed in order to plan effective action
Farchi 2008	Italy; TB	all population; immigrants, foreigners, foreign-born	See above in Misreporting	See above in Misreporting
Das 2006	New Zealand; TB	all population; ethnicity, place of birth	To describe the epidemiology of tuberculosis in New Zealand for the 10-year period 1995-2004.	TB is not declining in New Zealand. The burden of disease is very unevenly distributed across the population with marked ethnic inequalities.
Marc 2010	USA, HIV	>12 years old; Haitian-born adults and adolescents living in US	Describe HIV trends among Haitian-born persons living in the United States, and compare them to the US population and non Hispanic black ones. Identify the major risk factors associated with HIV morbidity in Haitian-born person.	Study findings show the importance of having accurate denominators to estimate rates of HIV for the Haitian population. Using estimates from the 2007 American Community Survey, results suggest a seven-fold over-representation of Haitians in the CDC AIDS surveillance data. In contrast, using denominator estimates from the Haitian Consulates, Haitian-born persons in the US, at this time, have a similar AIDS rates to blacks/African Americans overall, which challenges beliefs that Haitian immigrants have a higher prevalence of AIDS than other groups. Scientific methods need to be used to better understand what places Haitians at risk for HIV. Study authors recommend that research is urgently needed to adequately address prevention efforts for this ethnic group.
